# Systematic review of MCDM approach applied to the medical case studies of COVID-19: trends, bibliographic analysis, challenges, motivations, recommendations, and future directions

**DOI:** 10.1007/s40747-023-00972-1

**Published:** 2023-02-03

**Authors:** A. H. Alamoodi, B. B. Zaidan, O. S. Albahri, Salem Garfan, Ibraheem Y. Y. Ahmaro, R. T. Mohammed, A. A. Zaidan, Amelia Ritahani Ismail, A. S. Albahri, Fayiz Momani, Mohammed S. Al-Samarraay, Ali Najm Jasim, R.Q.Malik

**Affiliations:** 1 Faculty of Computing and Meta-Technology (FKMT), Universiti Pendidikan Sultan Idris (UPSI), Perak, Malaysia; 2 Future Technology Research Center, National Yunlin University of Science and Technology, 123 University Road, Section 3, Douliu, Yunlin 64002 Taiwan, ROC; 3 Computer Techniques Engineering Department, Mazaya University College, Nasiriyah, Iraq; 4 Department of Computing, Faculty of Arts, Computing and Creative Industry, Universiti Pendidikan Sultan Idris, Tanjung Malim, Malaysia; 5 Computer Science Department, College of Information Technology, Hebron University, Hebron, Palestine; 6 Department of Computing Science, Komar University of Science and Technology (KUST), Sulaymaniyah, Iraq; 7 SP Jain School of Global Management, Sydney, Australia; 8 Department of Computer Science, Kulliyyah of Information and Communication Technology, International Islamic University Malaysia, Kuala Lumpur, Malaysia; 9 Iraqi Commission for Computers and Informatics (ICCI), Baghdad, Iraq; 10 E-Business and Commerce Department, Faculty of Administrative and Financial Sciences, University of Petra, Amman, 961343 Jordan; 11 Foundation of Alshuhda, Baghdad, Iraq; 12 Medical Intrumentation Techniques Engineering Department, Al-Mustaqbal University College, Babylon, Iraq

**Keywords:** COVID-19, Data privacy, Federated learning, Monoclonal antibodies, Multi-criterion decision making, Treatment, كوفيد -19, خصوصية البيانات, التعلم الاتحادي, الأجسام المضادة وحيدة النسيلة, صنع القرار متعدد المعايير, علاج

## Abstract

When COVID-19 spread in China in December 2019, thousands of studies have focused on this pandemic. Each presents a unique perspective that reflects the pandemic’s main scientific disciplines. For example, social scientists are concerned with reducing the psychological impact on the human mental state especially during lockdown periods. Computer scientists focus on establishing fast and accurate computerized tools to assist in diagnosing, preventing, and recovering from the disease. Medical scientists and doctors, or the frontliners, are the main heroes who received, treated, and worked with the millions of cases at the expense of their own health. Some of them have continued to work even at the expense of their lives. All these studies enforce the multidisciplinary work where scientists from different academic disciplines (social, environmental, technological, etc.) join forces to produce research for beneficial outcomes during the crisis. One of the many branches is computer science along with its various technologies, including artificial intelligence, Internet of Things, big data, decision support systems (DSS), and many more. Among the most notable DSS utilization is those related to multicriterion decision making (MCDM), which is applied in various applications and across many contexts, including business, social, technological and medical. Owing to its importance in developing proper decision regimens and prevention strategies with precise judgment, it is deemed a noteworthy topic of extensive exploration, especially in the context of COVID-19-related medical applications. The present study is a comprehensive review of COVID-19-related medical case studies with MCDM using a systematic review protocol. PRISMA methodology is utilized to obtain a final set of (*n* = 35) articles from four major scientific databases (ScienceDirect, IEEE Xplore, Scopus, and Web of Science). The final set of articles is categorized into taxonomy comprising five groups: (1) diagnosis (*n* = 6), (2) safety (*n* = 11), (3) hospital (*n* = 8), (4) treatment (*n* = 4), and (5) review (*n* = 3). A bibliographic analysis is also presented on the basis of annual scientific production, country scientific production, co-occurrence, and co-authorship. A comprehensive discussion is also presented to discuss the main challenges, motivations, and recommendations in using MCDM research in COVID‐19-related medial case studies. Lastly, we identify critical research gaps with their corresponding solutions and detailed methodologies to serve as a guide for future directions. In conclusion, MCDM can be utilized in the medical field effectively to optimize the resources and make the best choices particularly during pandemics and natural disasters.

## Introduction

The earliest large-scale pandemic witnessed by people who may still be alive today was the Spanish flu (during World War 2) [1]. Since then, the preparation for pandemics has gained increased attention from researchers, governments, and decision makers [2]. However, when COVID-19 started spreading, humans knew that beyond any doubt that the preparations were not up to the event [3]. Tens of millions of people lost their jobs, and the world witnessed global financial crises, millions of death cases, and slow economic and health-system recovery [4]. The results of COVID-19 pandemic are different decisions, including long and/or long lockdowns [5], borders closing [6], quarantines [7], months/years of mask policy [8], offer financial aids [9], imposing vaccine policy [10], and many other decisions. They are not pre-planned for pandemic cases but have been decided during the evaluation of health and economic system by decision makers. Since then, researchers from all scientific disciplines have exerted immense effort in their respective areas of specializations [11]. Doctors and medical practitioners are not the only heroes during the pandemic; all researchers who have utilized their academic disciplines to provide assistance are also heroes [12]. Given that COVID-19 has affected many domains, particularly healthcare (HC), and left a knock-on effect on diagnoses and treatments [13], among other challenges, [14], people have become concerned with the future and strength of medical institutions should a similar pandemic hit us again in the future [15]. The World Health Organization (WHO) claims that evaluating and understanding pandemic-associated factors is crucial [16]. Globally, COVID-19 is acknowledged to have altered many aspects of daily human life, ranging from the medical area [17] to the social [18], economic [19], technological [20], and patient-related fields [21]. Additionally, COVID-19 has significantly impacted academics, specifically in terms of scientific studies on the different COVID-19 cases and factors [22]. Nevertheless, scientific research on COVID-19 related context can be turned into valuable insights to assist in the curb against the pandemics, and among the variable examples in that regard is the role of decision support systems, and others [23]. The involvement of expert systems includes optimization techniques [24] (to optimize limited resources during pandemics), artificial intelligence (AI; to predicting or analyzing different pandemics situations) [25], and decision making (DM; to make the best decision among several available options or analyzing different decisions) [26]. This involvement can help in better preparing against emerging epidemics. Technological advances, particularly those associated with AI and decision science, have relatively contributed in addressing the pandemic [27]. AI is one of the most emergent technologies, particularly when incorporated it to medicine-related decisions to fight COVID-19 [28–30]. Whether used for detection, control or any other purposes, technological advances have been assessed against this pandemic from different viewpoints [31]. Another associated emerging technology is the DM algorithm [32], which present various potentials and other views. Capacity evaluation of contributions for COVID-19 using multicriterion decision analysis (MCDA) techniques is extensively used to reveal the relationship among the evaluation criteria and select other alternative cases [33].

Multicriterion DM (MCDM) is considered among the most utilized tools in various COVID-19 related areas. MCDM can assist in providing different benefits in eliminating COVID-19 conditions and infected patients’ concerns [34]. Additionally, it can help in developing proper decision regimens, prevention strategies, and drug and vaccine development with precise judgment. Academic literature indicates that MCDM is utilized during the COVID-19 pandemic in various cases and aspects, particularly in terms of case studies or method enhancement. Case studies discuss various studies, in which MCDM was used in such areas as medical decision assistance and economic and financial aspects. Method enhancement refers to researchers pursuing theoretical MCDM enhancements and using enhanced methods in COVID-19 case studies. Nevertheless, additional investigation should be conducted in reviewing all MCDM and COVID-19 studies in terms of medical applications and identifying potential future research gaps. To the best of the authors’ knowledge, most MCDM and COVID-19-related studies have primarily present the role of MCDM in specific case studies, and no detailed reviews of medicine-related research have been conducted. Accordingly, focus must be directed on addressing current gaps based on the challenge of real treatment distribution and appropriate methodologies must be used in the future. The present study aimed to analyze the MCDM and COVID-19 medicine-related studies, present the current challenges and gaps, and propose a detailed methodology for future results. The contributions of this study are as follows.

- A comprehensive review and analysis of MCDM and COVID-19-related medicine case studies are conducted to classify academic literature into four categories: diagnosis, safety, hospital, and treatment.
- A bibliographic analysis was presented in the basis of annual scientific production, country scientific production, co-occurrence, and co-authorship.
- Different challenges, motivations and recommendations for COVID‐19 cases under MCDM theory are discussed.
- New emerging gaps are identified and a solution with detailed methodology is proposed.

## Systematic Literature Review (SLR) Protocol

This study is conducted using a systematic literature review (SLR) approach to help thoroughly understand the research topic and supplement subsequent investigations with extensive data [11]. Compared with traditional review methods, SLR is a well-structured procedure capable of improving research synthesis by identifying relevant papers based on selected parameters. SLR is also a cutting-edge method applicable to a wide variety of research domains and scientific disciplines. It entails several key steps, including scope identification, search-mechanism development, study selection and extraction, and information synthesis. Given the advantages of SLR, it is adopted in this research to study MCDM utilization in medical settings during the COVID-19 pandemic. The following subsections elaborate on the SLR subprocesses.

### Information source

This study collected information based on the strategically search method following the SLR and meta-analysis (PRISMA) phases. To collect information, five scientific database search engines are utilized to search, filter, extract, and draft this survey: (1) ScienceDirect, which contains wide high-impact studies in various domains; (2) Web of Science, which includes numerous scientific research publications in different fields; (3) IEEE Xplore, which provides access to numerous scientific studies from multidisciplinary technologies in numerous domains; and (4) Scopus. The selected databases are considered most suitable and adequate for this review owing to their scientific soundness and academic resilience by including various SLR publications in high-impact scientific journals.

### Search strategy

The search was conducted on 28 June 2020 and updated on August 2022. The most recent studies are included by performing another search in August 2022 in the advanced search boxes of the aforementioned databases. Boolean operators (e.g., OR and AND) are utilized for the search to combine the different synonyms with the two groups of keywords in the process. In searching and filtration, different types of publications, such as conference papers, research and review articles, are selected.

### Study selection

**Fig. 1:**
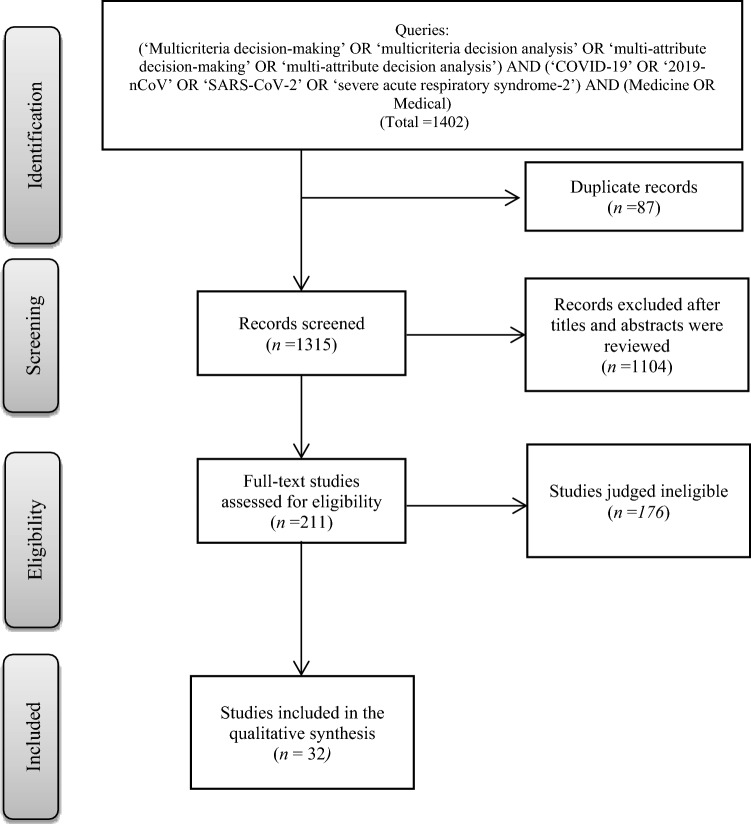
SLR protocol

The study selection procedure is performed through three steps, as presented in Fig. 1. First, all articles found in the search are collected initially and scanned thereafter to remove duplicates. The initial number of collected articles is 1402, 87 of which are duplicates. Second, the titles and abstracts of the extracted articles are scanned based on our inclusion and exclusion criteria to identify the relevant articles and be included in the final round. Third, full-text reading is conducted for each article that matches our inclusion criteria to extract valuable information and be analyzed for the review. Accordingly, 1104 articles not matching our inclusion criteria and are excluded. The final set of relevant articles for this review comprises 35 articles.

### Inclusion and exclusion criteria

We define a set of inclusion and exclusion criteria to identify the related articles during the study selection. Articles are included if they are research or review articles, published in the English language, and discuss the utilization of MCDM techniques under the medical field during the COVID-19 pandemic. By contrast, articles are excluded if any of the preceding criteria is not identified.

### Discussion flow

**Fig. 2:**
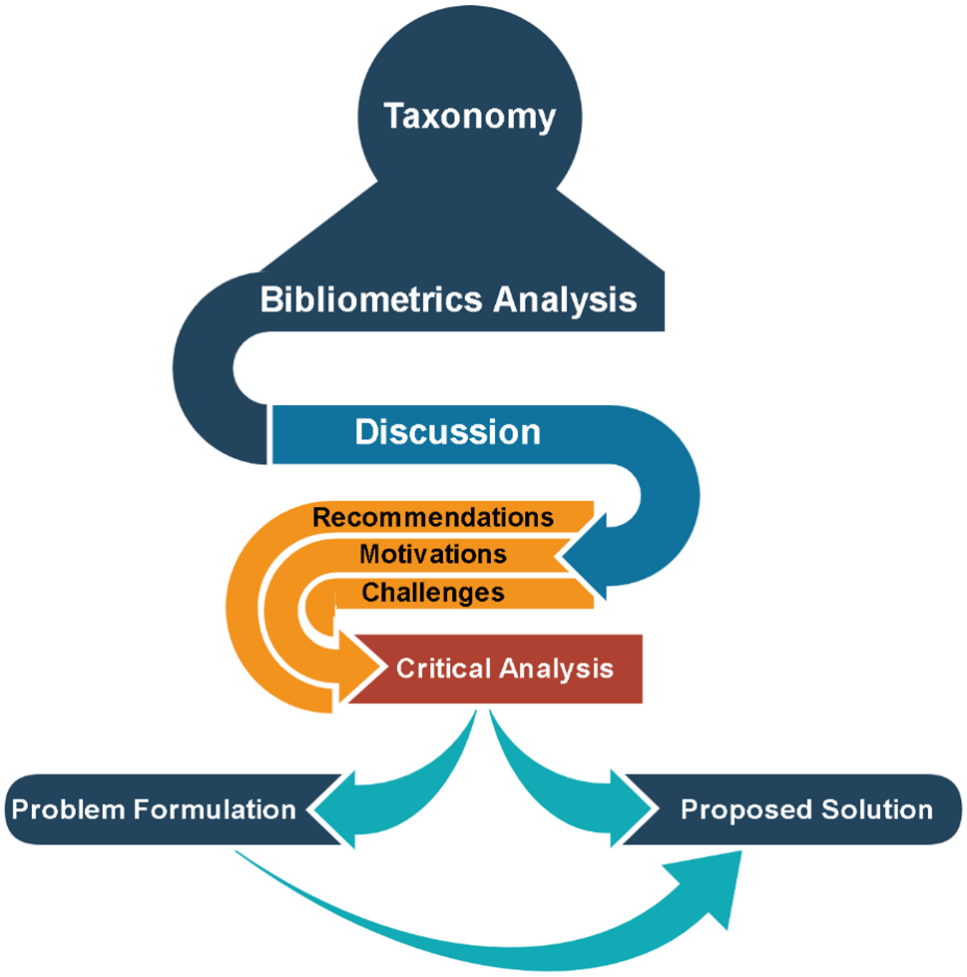
Discussion flow

The process of this systematic review begins with the standard protocol items. Afterwards, the main layouts of this work are presented, showing how the flow of the discussion is presented (Fig. 2).

As shown in Fig. 2, the flow starts with a taxonomy analysis of all the MCDM medical-related research. Afterwards, a discussion is presented to present the main highlights, including challenges faced in previous works, motivations and recommendations. All these three aspects are utilized in defining the current research gap. Accordingly, the research gap is presented in two stages; the first to discuss the problem formulation, and the second to discuss the proposed solution.

## Comprehensive taxonomy

**Fig. 3:**
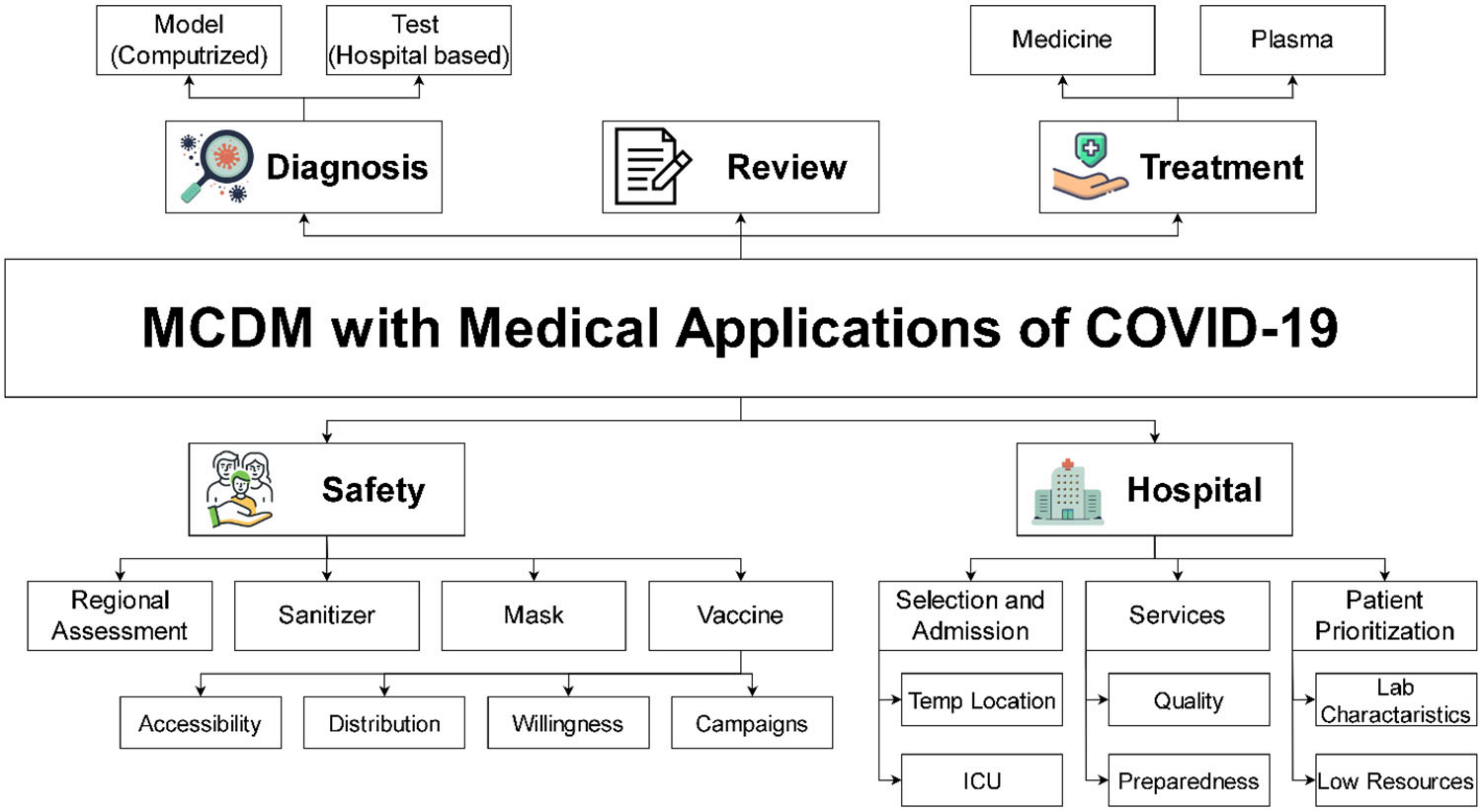
Taxonomy categorization

While scanning literature, articles are grouped in accordance with their medical perspectives. Given that this paper is an SLR, which combines decision science represented by MCDM and the medical applications of COVID-19, five main categories in the form of taxonomy are represented. The first category discusses how MCDM is integrated in such areas as COVID-19 diagnosis, whereas the second category discusses the safety aspect. The third category called “hospital” primarily discusses patients’ admission to hospitals and their prioritization during the COVID-19 pandemic. The fourth category discusses treatment through prescribed medicine and plasma transfusion. The last category is called “reviews” and primarily discusses and presents other SLR or reviews discussing MCDM and COVID-19 studies. At the end of the section, a critical point of view presents the differences of this SLR with that in previous research published. The design of the taxonomy in this form has been agreed upon among the authors and in consideration of their main observations of literature. Therefore, this presentation of the taxonomy enables a more comprehensive understanding of the topic, as shown in Fig. 3.

### Diagnosis

The first category has two main subcategories: (1) research discussing diagnosis through computerized and hospital-based tests and (2) studies related to safety measures, such as regional assessment, masks and sanitizer. Two main themes are observed. The first by [35] utilizes MCDM in identifying key indicators from initial blood routine test results to predict COVID-19. The technique for order of preference by similarity to ideal solution (TOPSIS) and machine learning (ML) classifiers are subsequently used to further select effective indicators from patients’ initial blood-test results, which are later used in the prediction. TOPSIS is also used with the best–worst method (BWM) in Ref. [36], in which an MCDM model is constructed to distinguish COVID-19 from four other viral chest infections in an uncertain environment by utilizing the viruses’ primary symptoms and CT scans. The authors re-use TOPSIS in Ref. [37], in which a spherical intelligent fuzzy-decision model is developed to diagnose COVID-19 and control emergency situations to choose the best path to overcome this deadly disease. Authors in Ref. [38] used complex fuzzy (CF) sets and developed an MCDM Dombi operations-supported model for diagnosing COVID-19. Another CF set called m-polar neutrosophic set is utilized to diagnose COVID-19 in Ref. [39]. Lastly, [40] used the TOPSIS and entropy approaches in discussing an MCDM methodology for the problem of evaluation and benchmarking of COVID diagnosis ML models.

### Safety

In the safety subcategory, four main safety aspects are discussed linking MCDM in medical settings with regional assessment, sanitizers, mask selection, and vaccine. For regional assessment, [41] presented a comparative research among well-known MCDM methods, including TOPSIS, VIKOR, and complex proportional assessment. A total of 100 world regions are utilized to evaluate and analyze the safety levels for COVID-19 in these regions. Other remaining studies in the safety subcategory includes Ref. [42], which presents a new MCDM approach based on a Pythagorean fuzzy soft-set environment. The approach presents its capability in dealing with unsatisfactory data, ambiguity, and inconsistency presented in the MCDM context. The aforementioned research is used to select effective hand sanitizers during the COVID-19 pandemic, and the approach is compared with others in terms of effectiveness. Two other studies in this category have focused solely on the selection of antivirus masks during the COVID-19 pandemic. The first study is Ref. [43], the main aim of which is maximizing the usage of anti-virus masks while using the MCDM approach presented over a spherical normal fuzzy (SpNoF) set. The second research is Ref. [44], which presents the same application over q-rung orthopair uncertain linguistic sets. Four topics are discussed for the vaccine. The first topic is vaccine accessibility, discussed by [45] concerning vaccination centers, particularly in the case of disasters and pandemics (e.g., COVID-19). For the second topic, Ref. [46] discussed the issue of vaccine distribution using AHP and TOPSIS for COVID-19 vaccine alternatives. The authors use the MCDM approaches, and their findings suggest that HC personnel, people with pre-existing medical conditions, the elderly, essential employees, and pregnant and breastfeeding mothers are the most prioritized groups to receive the vaccines first. The next three studies utilize MCDM methodologies, including fuzzy-weighted zero-inconsistency (FWZIC) and fuzzy decision by opinion score method (FDOSM) for prioritizing COVID-19 vaccine dose recipients. The first attempt is conducted using Pythagorean fuzzy set [47], followed by T-spherical fuzzy environment [48] and q-rung orthopair fuzzy set (q-ROFS) [49]. The topic on vaccine willingness is presented in Ref. [50], which states that vaccination willingness is a significant concern. In this context, AHP is used as an MCDM strategy to ascertain the public’s willingness to receive COVID-19 vaccines. These findings suggest that individual choice, vaccine origin, adjusting to change, and perceived barriers to vaccination are the primary determinants of the willingness to receive COVID-19 vaccines. The most recent research presented in the vaccine category is Ref. [51], which uses q-ROFSs and VIKOR for applications in mass-vaccination campaigns in the COVID-19 context.

### Hospital

The hospital category discusses academic literature focusing on the use of MCDM in medical settings for COVID-19 in terms of (1) hospital selection and admission enabled by MCDM in various settings, (2) hospital services and (3) patient prioritization. For hospital selection and admission, a total of (*n* = 3) related studies are reported. The first study [52] utilizes the MCDM approach with the use of the BWM method in choosing the right place to build temporary hospitals as one of the most important and urgent measures for pandemic response. Meanwhile, Ref. [53] develops an emergency MCDM decision-support model based on spherical hesitant fuzzy for patient care and admission scheduling (PCAS). Lastly, selection and admission research by [54] elaborates on how the pandemic has resulted in an intense flow of patients to hospitals, particularly intensive care units (ICUs). This situation presents a challenge in admission decisions, in which MCDM is introduced to address the aforementioned challenge by prioritizing the admission criteria using AHP. Thereafter, a multiobjective optimization approach is used to rank COVID-19 patients. The following theme reports on hospital services regarding their quality and preparedness. Two main studies are presented. First, Ref. [55] studies prioritization factors contributing to hospitals’ quality of service from the viewpoint of patients and their companions during the COVID-19 pandemic. Accordingly, a hybrid MCDM approach is used in the process of using fuzzy AHP and PROMETHEE for hospitals ranking under normal conditions and during the pandemic. Second, a hospital services study used three MCDM methodologies, namely, FAHP, fuzzy DM trial and evaluation laboratory (FDEMATEL), and TOPSIS. A previous study [56] has evaluated and ranked the disaster preparedness of hospitals. The next topic discusses MCDM utilization in hospital settings during the COVID-19 pandemic for patient prioritization-related research. Three studies are presented. First, Ref. [57] used TOPSIS in patient prioritization while using multilaboratory criteria and patient data set, particularly for asymptomatic COVID-19 carriers. Second, Ref. [58] conducts patient prioritization based on biological laboratory examination criteria and COVID-19 patients’ list. The two MCDM techniques, namely, AHP and VIKOR, are used in the process. Lastly, Ref. [59] utilizes MCDM in medical settings in prioritizing non-critical COVID-19 patients in HC settings with limited resources.

### Treatment

The two main topics discussed for treatment-related studies are medicine and plasma. The first topic discusses medicine treatment for COVID-19 through two studies. First, Ref. [60] develops an MCDM approach extended over hesitant fuzzy sets to treat mild COVID-19 symptoms. The study is primarily for drug selection for COVID-19, and demonstrated practicability and efficacy in real-life applications. Second, Ref. [61] presents a close case study for medicine selection for patients with mild COVID-19 symptoms. The authors clarify the usage of the MULTIMOORA method because it is specific with the peculiarities of three subordinate models. The final topic in the treatment section discusses utilizing plasma in the COVID-19 and MCDM contexts. First, Ref. [62] suggests that patients recovering from COVID-19 have antibodies circulating in their blood, which, if given to deteriorating patients, may conceivably assist in enhancing their immune system. Thus, a hybrid methodology proposes to utilize ML and unique MCDM methodologies to present a rescue framework for the transfusion of the best convalescent plasma (CP) to the most critically ill patients with COVID-19 based on biological needs. Second, Ref. [63] provides CP to the most vulnerable patients, preventing the virus from spreading and healing those infected. The distinction between this effort and others is that the former is recommended on the basis of a centralized/decentralized telemedicine environment to provide CP from eligible donors to patients who are most in need.

### Review

This category discussed the review of related research in this taxonomy section in relation to MCDM studies related to medicine. Three studies are presented. First, Ref. [64] presents a systematic review for detection and classification of COVID-19 medical images in terms of evaluation and benchmarking. The review also proposes a detailed MCDM methodology for evaluating and benchmarking AI techniques used in all classification tasks of COVID-19 medical images. This method serves as a future direction on the basis of the integrated AHP and VIKOR methods. Second, Ref. [65] discusses the utilization of MCDM in the fight against COVID-19 in various applications, including medical, social, and technological. Accordingly, the authors perform interesting patterns of analysis that can assist future studies in the area, including MCDM-COVID-19 challenges, contributions, and bibliographic analysis. Lastly, Ref. [66] discussed the MCDM utilization for treating COVID-10, with theoretical analysis discussing all aspects in applying MCDM in COVID-19-related studies. Several interesting topics covered in this paper include standardizing the assessment criteria, applying MCDM theory to rank and weigh alternative evaluation criteria, normalizing data utilized in the study, MCDM theory contexts, and selecting experts and validation methodology for effective MCDM theory. The authors conclude with a suggested future direction to provide scholars and the broader community with an overview of the current state of MCDM assessment and development methodologies that can be used to harness MCDM potentials in combating COVID-19. By recognizing the preceding medical cases, none of them are clearly presented in the manner by which this study is formed. The first review [64] is concerned only with the identification and detection part, and MCDM is part of the entire process. However, it included others such as ML, and is concerned only with medical image, evaluation, and benchmarking. For the second review [65], MCDM is reviewed in relation to numerous cases, among them is the medical one. However, as the authors conduct their research, detailed explanation reflecting a real case-study challenge with the proposed methodology is not presented, which is the main focus of the current study. In Ref. [66], they are concerned with the theoretical development of MCDM in various COVID-19 cases. However, a detailed medical discussion is not presented, let alone a real challenge concerning medical case studies of treatment by using the proposed methodology, which is also the main discussion and idea of the current review.

## Bibliometric analysis

The rapid increase in publications and ongoing research has increased the challenge of keeping up with empirical contributions and enormous research streams. Hence, accumulating decisive evidence from previous studies is becoming increasingly complicated. Accordingly, the SLRs aim to summarize the results of literature, formulate problems, and propose solutions accordingly. The reviews also provide compilation of literature results, broadening the knowledge base, identifying theoretical, practical, and methodological gaps, and enhancing the research plan. However, the issue of reliability and objectivity in systematic reviews remains a challenge that needs to be resolved. To address the issue of reliability and transparency, numerous ongoing studies suggest quantitative and qualitative approaches that enhance systematic reviews by reorganizing the findings of literature. Among the quantitative and qualitative tools, bibliometrics based on R-tool and VOSviewer is considered the most reliable and transparent method [109]. The bibliometric method provides organized results, summarizes research trends, explores prolific countries, institutions, and authors, thereby presenting the big picture of extant research. Bibliometrics based on R-tool and VOSviewer is not cumbersome for researchers to use, does not require professional, and is open source to perform comprehensive bibliometric analyzes. Furthermore, this study adopted bibliometrics based on R-tool and VOSviewer to explore annual scientific production, country scientific production, co-occurrence, and co-authorship.

### Annual scientific production

**Fig. 4:**
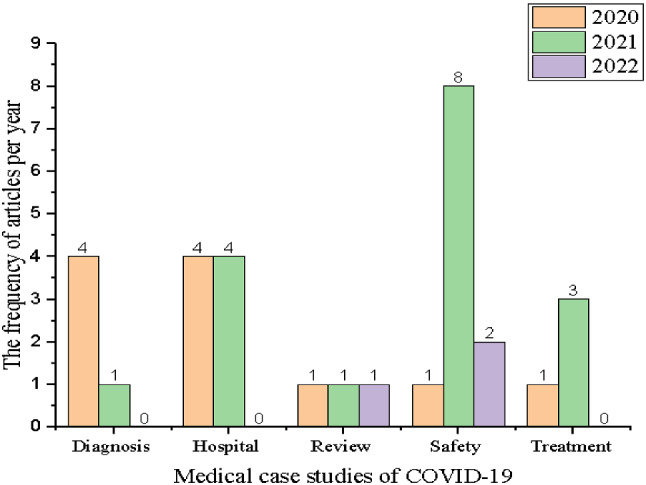
Annual scientific production

The scientific production of medical case studies of COVID-19 based on the MCDM approach is developing at a rapid pace. To identify the applicable methodological results, the annual scientific production aims to describe the structure of scientific disciplines and research developed over time. Figure 4 shows the historical development of publications for medical case studies of COVID-19 through the MCDM approach.

As illustrated in Fig. 4, the most productive category over the years is safety. COVID-19 created a danger to human life and caused a complete closure of many countries. Various academics and practitioners have focused on the topic of safety to mitigate the risk of COVID-19. The years 2021 and 2022 have seen an increase in academic publications that develop medical protective tools such as masks and gloves. However, hospitals and health centers faced the challenge of the enormous increase in the number of patients, especially in 2021. Consequently, the scientific production of the hospital category for the years 2020 and 2021 rose to improve the level of quality and treat patients according to the priorities of the health condition. At the beginning of the epidemic’s spread, the interest in identifying methods of diagnosing the virus and controlling emergency situations increased. Such increase in the academic scientific of the diagnosis category was high in the year 2020. Moreover, the risks of the spread of the COVID-19 pandemic have overwhelmed academics and practitioners to mitigate the pandemic. In the year 2021, attempts to eliminate the epidemic increased. Hence, the scientific publications in 2021 for the treatment category were high. For the review category, the attempts were minor for the last three years. Thus, the current study pursues to address the literature gap by providing a comprehensive and insightful view of ongoing and future research.

### Country scientific production

**Fig. 5:**
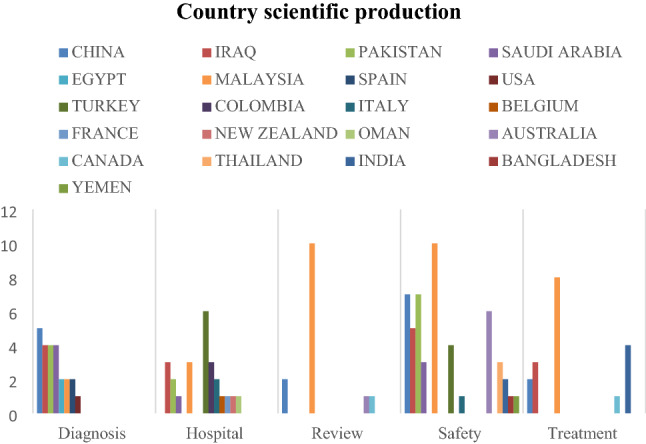
Country scientific production

COVID-19 has affected all countries. Governments have responded in various ways to mitigate the threat of such a disease to life. Country scientific production describes the scientific publications of medical case studies of COVID-19 through the MCDM approach at the country level. Figure 5 demonstrates country scientific production to provide an insight for practitioners and academics regarding the most effective category in crises and pandemics.

The safety category is receiving extensive attention from literature in all countries because countries and organizations have implemented work from home to reduce the risks of interaction and spread of COVID-19 infection. Bibliometric analysis of literature confirms that Malaysia is the most productive country for studies using MCDM with medical aspects in the safety, review, and treatment category, followed by China for the treatment, safety, review, and diagnosis category. Nevertheless, Turkey is interested in improving the quality of health services and is the highest contribution at the level of the hospital category. Analysis of country scientific production delivers practitioners with insight into identifying the most interesting countries in improving HC services and developing medical protection tools. Moreover, governments can benefit from the success stories of countries to overcome future crises. Identification of practitioners for countries that have failed in facing the epidemic at the level of treatment, safety, review, diagnosis, and hospital category contributes to addressing the most important challenges and issues that the medical system suffers from in such countries. Country scientific production also provides guidance for academics to address theoretical and practical gaps in literature on the basis of country application.

### Co-occurrence

**Fig. 6:**
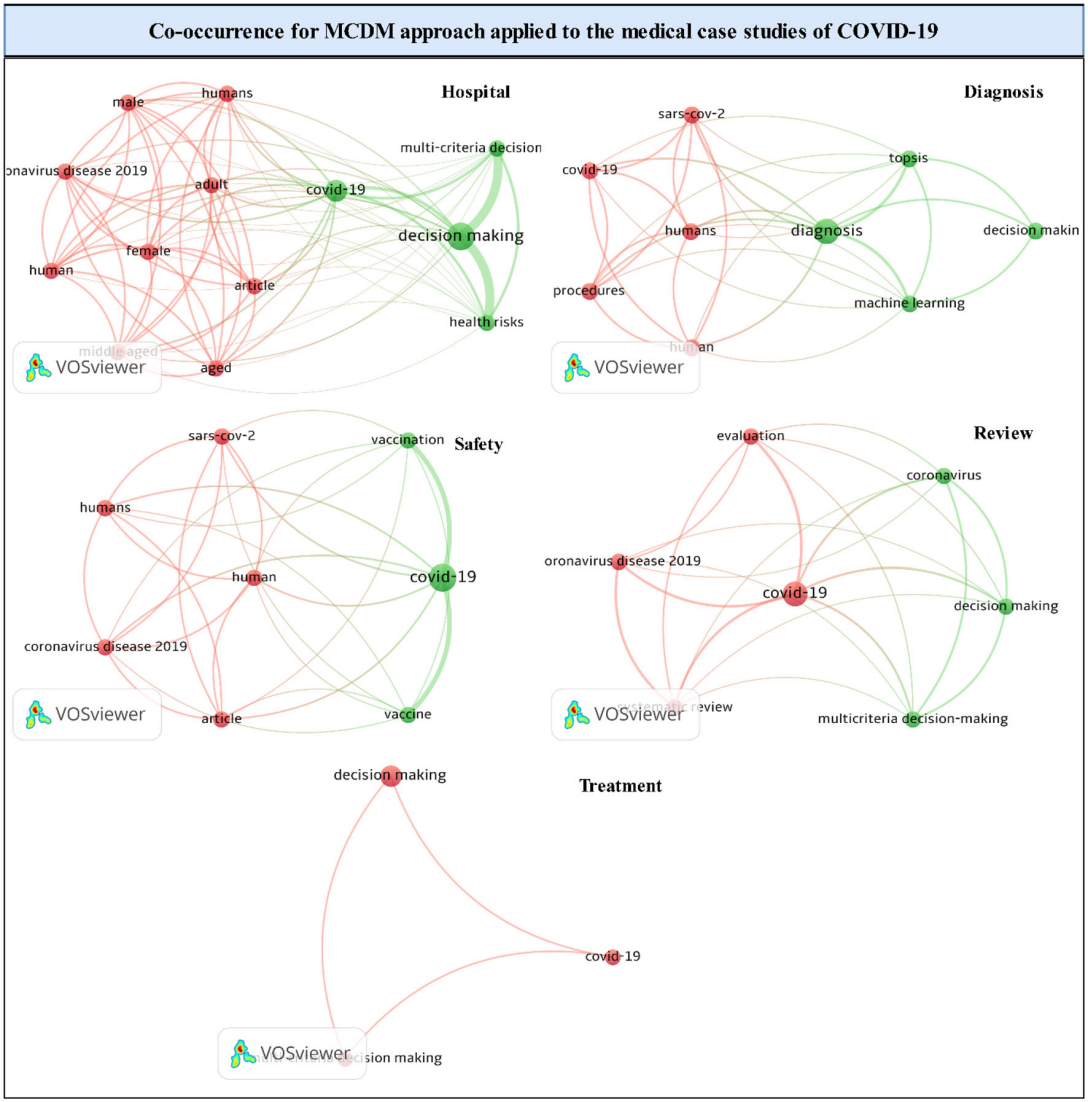
Co-occurrence

Co-occurrence is a methodology containing common keywords introduced by literature. Co-occurrence analysis maps the conceptual structure of a field. Such an analysis is referred to as a semantic network. Hence, academics and practitioners identify the relationship of the main topic with the emerging subfields. This analysis depicts the frequency of occurrence of keywords. Figure 6 presents co-occurrence analysis for the diagnosis, safety, hospital, treatment, and review category.

For the diagnosis category, ML and DM are the most frequent topics. Increasing the thickness of the line separating two keywords indicates the close relationship between the keywords of diagnosis category. For safety categories, previous literature has investigated the impact of COVID-19 on gender and age based on the use of the MCDM approach. However, the review category indicates that literature is interested in reviewing the mechanism of application of MCDM methods with COVID-19. Additionally, the vaccine is the most important common issue addressed in the safety category. Co-occurrence analysis indicates DM is a vital method to prioritize COVID-19 patients and distributing plasma for them. By capturing the co-occurrence of keywords for medical case studies of COVID-19, practitioners and academics can use information networks to facilitate efforts to reorganize the available information and findings. Furthermore, stakeholders could identify the intellectual base of MCDM approach applied with medical case studies of COVID-19.

### Co-authorship

**Fig. 7:**
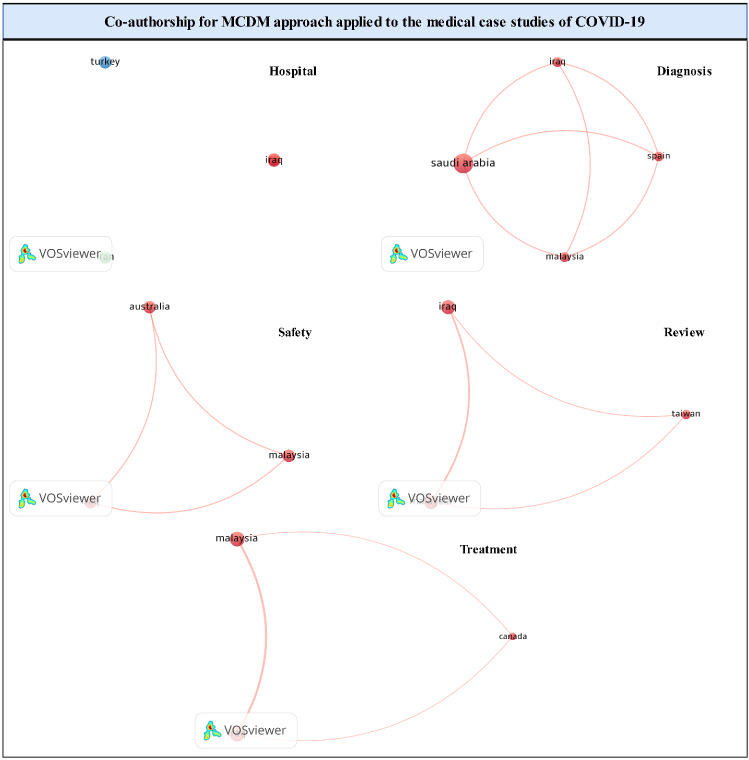
Co-authorship

Co-authorship networks are defined as scientific publications by a group of scholars. Co-authorship analysis contributes to identifying networks of cooperation among countries, institutions, and authors. Such an analysis determines the number of papers written by researchers, identify the patterns of cooperation between researchers, and explore the number of co-authors. Figure 7 shows the results of a co-authorship analysis of medical case studies of COVID-19.

The node size represents the number of articles, the nodes represent authors, and the lines show the pattern of co-authorship. For the diagnosis category, scientific cooperation exists among Iraq, Malaysia, Spain, and Saudi Arabia to mitigate the risks of COVID-19 and benefit from the experiences of European and Asian governments. However, the hospital category does not witness scientific cooperation owing to the different ways of admitting, exiting, and treating COVID-19 patients between countries. Scientific cooperation exists among Malaysia, Iraq, Australia, and Canada at the level of review, safety, and treatment categories. In this context, Malaysia and Iraq represent the main players in international cooperation, followed by Australia and Canada, in descending order. Identification of scientific cooperation regarding the application of the MCDM approach with medical aspects enables practitioners to maximize the outcomes of resources and contributes to maximizing impact for academics by increasing citations. Scientific cooperation contributes to increasing the learning of new skills and increasing the chances of solving problems on a strong scientific basis. However, the co-authorship analysis confirms the weakness of scientific cooperation in applying the MCDM approach with medical aspects of COVID-19. Moreover, weak scientific cooperation reduces the chances of career expansion and science promotion.

## Challenges

**Fig. 8:**
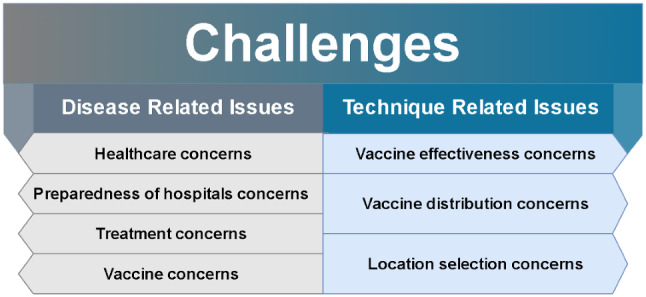
Main challenges

Challenges refer to issues and gaps identified and encountered by researchers from previous scientific studies, addressing them openly for new potential research in the area and promoting future ideas. For this review, challenges are discussed in relation to a disease (i.e., COVID-19) and MCDM techniques, as shown in Fig. 8. The details are as follows.

### Disease-related issues

The COVID-19 pandemic has posed a serious threat to society and the economy, eventually exploding into a public health crisis. An increasing number of academics have noted data and understanding gaps in relation to how the COVID-19 pandemic spreads within and between communities, as well as its potential effects on vulnerable and general populations [67]. Furthermore, COVID-19’s rapid spread globally poses a significant threat to public safety and potential therapy. Some COVID-19 symptoms are similar to those of other viral chest disorders, resulting in difficult creation of models for effective COVID-19 detection [36]. The proliferation of COVID-19 has likewise resulted in extensive economic and social damages. COVID-19 affects the majority of population groups, but it is most dangerous to the elderly, people with disabilities, those living in poverty, homeless people, refugees, migrants, youth, and indigenous peoples [51]. Evidently, addressing this challenge appropriately constitutes substantial difficulties. Meanwhile, identifying mild from serious health cases is a difficult but crucial task, particularly when the medical sector offers HC services, especially because these services incorporates treatment aspects [58]. The management of COVID-19 spread in crisis situations is challenge [37]. Additionally, national and global health organizations (e.g., WHO, UNICEF, and CDC) have consistently recommended national governments to use various measures to curb the spread of COVID-19 and devise effective treatment protocols and strategies [68, 69]. However, national treatment and immunization programs must be trained to elucidate global guidelines and make decisions best suited to their context.

#### Healthcare concerns

The spread of COVID-19 has prompted governments to establish national policies aimed at mitigating the disease’s burden on HC systems [70] and developing proper treatment strategies. The scarcity of resources and endless demands restrict HC services to be supplied to all individuals in need. This situation became increasingly evident during the COVID-19 pandemic [71]. The COVID-19 pandemic has placed a significant burden on global HC systems, with developing countries bearing the brunt of the damage owing to their underdeveloped HC infrastructure and limited financial resources [72]. Hospitals’ emergency reaction to HC management lacks sufficient systems for providing HC to COVID-19 patients [53]. Additionally, the COVID-19 pandemic has strained emergency response systems globally, resulting in the breakdown of health systems, law enforcement, and first responders [73], as well as decentralized hospital administration factors, such as scalability and management challenges for concurrently prioritizing COVID-19 patients and donors [63]. The preceding aspects further pose a significant threat to the health and lives of people globally, thereby providing exceptional strain to medical systems [35]. Hence, building the resilience of HC systems has become essential.

#### Preparedness of hospital concerns

As the number of COVID-19 patients increases, several health structures are impacted, including the requirement for additional beds and ventilators. Hospitals must anticipate the impact of COVID-19 on all sectors and work collaboratively to share knowledge and resources to guarantee optimal care. This situation contributes to good administration by ensuring the inclusion and care of all HC workers and organization of contact with the general public [53]. Similarly, a dearth of adequate hospital beds and ICU facilities for severely ill patients have been important issues [59]. In prioritizing ICU admissions for particular patients, guidelines based on a scientific approach should be developed to determine which COVID-19 patients should be prioritized for ICU admission or treatment in emergency or resource-constrained situations [54]. Consequently, the demand for operating room efficiency has a significant impact on hospitals’ financial and final ethical outcomes. Another crucial issue is that with the unexpected occurrence of natural and man-made disasters globally, the relevance of hospital preparation becomes evident. Notably, hospital preparation is the first point of contact for individuals seeking HC services. Hence, determining the level of disaster readiness of hospitals is a critical problem [56]. Additionally, no specific guidelines have been formulated for prioritizing COVID-19 patients for hospital admission in situations with significant bed shortage [59]. Accordingly, selecting the best location for temporary hospitals is one of the most crucial and urgent pandemic-response measures [52].

#### Treatment concerns

Patients with COVID-19 exhibit wide-ranging symptoms during the course of their illness, thereby creating an uncertain situation, in which doctors are unsure of the most effective medication. The medical community remains concerned with the absence of a standard therapeutic protocol leading to variability in the management of COVID-19 patients [74]. Accordingly, a realistic way to increase the efficiency of treatment and optimize the allocation of medical resources is needed. Another issue is determining the best way to distribute vaccines on a local and global scale. The issues of procurement, storage and distribution of vaccines and other therapeutic alternatives must be regulated. Additionally, rules should be formulated and a timeframe must be provided for assigning the generally accessible COVID-19 therapy choices for distinct priority groups.

#### Vaccine concerns

COVID-19 vaccines are supposed to protect humans against COVID-19. However, many individuals are on the fence regarding whether or not they should be vaccinated [50]. Considerably limited public trust in vaccines, resulting in poor vaccination uptake, continues to be a critical issue for European Union policy makers [75]. Serious side effects have not been encountered in COVID-19 vaccine clinical trials and current vaccination endeavors. Post-vaccination side effects are often mild [76]. Clinical signs are not observed in either clinical trials or current immunization undertakings. Post-vaccination side effects are often minor [77]. However, vaccination research should be conducted to address low vaccine confidence and uptake.

### Technique issues

From a theoretical standpoint, the increase in MCDM in addressing COVID-19 requires deeper examination to fulfill the main characteristic factors that fit in merging MCDM and COVID-19 [66]. HC restriction has consistently been present and unavoidable, and the HC industry is impacted by numerous COVID-19 patients. A solution is urgently required to prevent the danger of deteriorating patient health in terms of prioritizing based on their health conditions. Owing to (1) numerous biological laboratory screening criteria, (2) criteria weight, and (3) trade-offs between criteria, prioritized COVID-19 patients considered a complicated MCDA issue [58]. Additionally, prioritizing access to care treatment during extremely difficult times is needed [59]. Determining priorities is a difficult and seemingly unsolvable problem because it includes balancing efficiency with equality across several different criteria [71].

#### Vaccine effectiveness concerns

Various vaccines have been developed and are currently being evaluated by health authorities globally. However, numerous countries, particularly those with minimal resources, have significant difficulties in ensuring widespread access to vaccines [68]. Several firms are also attempting to correctly distribute vaccines and treatments, but it is bound to be difficult to implement for approximately 8 billion people. Consequently, competition has emerged, and competitiveness is becoming considerably fierce. Hence, governments must firstly select priority groups for providing COVID-19 vaccine doses [46]. The final solution to halt the COVID-19 pandemic is the development of effective vaccines. Experts and governments agree that vaccines may not be ready for another 2 years, but this situation does not imply that it cannot become available earlier [36]. Accordingly, an efficient and immediate protection technique must be developed before vaccines are produced.

#### Vaccine-distribution concerns

The difficulty of the MCDM issue is highlighted in the distribution of COVID-19 vaccines, so strong and durable MCDM approaches are needed [48]. Governments must adopt a priority system for distributing COVID-19 vaccine doses throughout the populace and prevent their random distribution [48]. Authorities must create a prioritized approach for allocating COVID-19 vaccine doses to people and avoid randomization of vaccine distribution [48]. Evidently, choosing the best vaccines and other treatments for target populations is critical [76].

#### Location selection concerns

MCDM is infrequently applied in analyzing government intervention strategies against COVID-19. Furthermore, MCDM difficulties are exacerbated by imprecision and unpredictability [78]. Government solutions are evaluated using several contradictory criteria, including cost effectiveness, simplicity of implementation and efficacy in limiting COVID-19 transmission [67]. Additionally, selective state action during the COVID-19 pandemic is an MCDM issue in a hazy and unclear environment. Governments and medical communities shift their priorities in response to emerging difficulties, and the success of interventions implemented in various countries [78]. Site selection of vaccination centers is a major challenge for the health sector [45]. For the hospital-site selection issue, various MCDM studies have been conducted but none of them has proposed an all-encompassing set of criteria for addressing this issue [52]. Inadequate sensitivity in clinical diagnostic procedures is one of the primary causes of the rapid spread of COVID-19 in communities [64]. Therefore, effective and immediate methods for hospital location selection are needed.

## Motivations

**Fig. 9:**
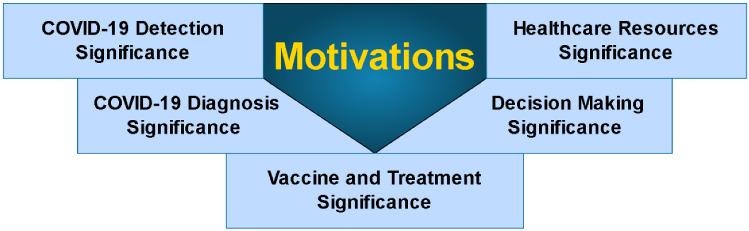
Main motivations

Reviewing and analyzing MCDM and COVID-19 motivate researchers, decision makers, and governments because of the evident and compelling benefits. This section is organized into five categories: COVID-19 diagnosis, DM, HC resources, vaccines/treatment and COVID-19 detection. Motivations in relation to the COVID-19 pandemic are discussed (Fig. 9).

### COVID-19 diagnosis significance

Scientists have been investigating various strategies to combat the COVID-19 pandemic since the first cases were discovered among the general population. Accordingly, they have identified the critical roles played by numerous scientific fields throughout this pandemic. MCDM has been implemented extensively in our everyday lives in many ways with several successes stories to aid in analyzing complicated problems and delivering an accurate DM process [66]. MCDM is used to solve health problems faced by workers in various environments [79]. It assists governments in developing the optimal strategy because a better grasp of methods is essential in battling the pandemic [78]. MCDM may also be used to assess and benchmark the various diagnostic models for COVID-19 in terms of the evaluation criteria [40]. The purpose of a diagnosis assistant is to classify patients as confirmed, suspected or suspicion of COVID-19 infection. A diagnosis assistant also classifies patients as mild, moderate, severe or critical [80].

### Decision-making significance

For medical specialists, MCDM may be utilized to resolve and mitigate the aforementioned complexities because it is a verified and robust scientific process that produces exact and effective outcomes in important situations of MCDM [74]. Additionally, it assists decision makers in achieving an effective outcome by enabling the evaluation of all possible elements affecting the choice issue [41, 81]. Prioritizing options for complete catastrophe preparation and response is rendered easier with MCDM use [78]. A DM tool has been established to address the issue of defining priorities among patients contending for minimal health facilities [71, 82]. Consequently, providing decision assistance may aid in improving decision quality, reliability, and clarity.

### Healthcare-resource significance

MCDM approaches have been proven effective in numerous HC data-focused applications [36]. A resilient HC sector can address any type of emergencies in a country [70, 72]. It is capable of identifying priority locations, comparing populations, and providing alternatives for government agencies to respond. Hospitals are among the most significant HC facilities because it is the primary provider of HC services, particularly during a pandemic, such as COVID-19 [55]. Hospitals often lack medical equipment and manpower [53, 56], so identifying hospitals with a poor degree of preparation is critical for disaster preparedness planning. Additionally, hospitals should plan for physical infrastructure and resource allocation because they are the only locations that can offer immediate treatment in the event of a crisis [56]. Undoubtedly, hospitals can mitigate the harm caused by disasters by arranging hospital resources (e.g., treatment areas, equipment and employees) and determining their disaster readiness levels. MCDM methods may aid in addressing hospital-site selection because to their intrinsic benefits than other OR methodologies, such as their capacity to include qualitative criteria together with quantitative ones [52]. Beneficial PCAS that is successful and precise has a good impact on hospitals in terms of service availability, management quality, affordability and social impact, among others [53]. Prioritizing infected individuals for treatment and distinguishing their important health issues are advantageous and could aid hospitalization worries in detecting health disorders [57]. By implementing an active risk management strategy, illnesses may be prevented and beneficial organizational improvements can be facilitated [82], thereby decreasing mortality and hospitalization costs.

### Vaccines and treatment significance

Vaccines are a significantly effective means to protect people against deadly diseases and save millions of lives. Public-health officials and multiple countries have shown positive indications on COVID-19 vaccines and treatment, which are intended to protect the public against severe COVID-19 [50]. Various researchers have been working on finding an effective single course of COVID-19 treatment [74]. Medical professionals and pharmacologists are also working relentlessly to identify and prescribe a standardized and effective course of treatment for COVID-19 patients [74]. Similarly, COVID-19 vaccine studies have been conducted by numerous governments/manufacturers using various methods [45]. Numerous health organizations have been working with biopharmaceutical companies to accelerate the process of finding a drug or vaccine for novel coronavirus [68]. WHO and other governing bodies have formulated guidelines and remedial activities to minimize the spread of COVID-19. As the death ratio caused by COVID-19 increases, the most suitable vaccine for people should be selected [76], and the best approaches for distributing such vaccines and other treatment forms must also be established [77]. Mass vaccination campaigns (MVCs) are implemented to control and remove infectious diseases in large populations [45]. MVC implementation encourages the prevention and control from vaccine-preventable diseases and high-impact diseases [51].

### COVID-19 detection significance

The use of AI and ML is expanding in different fields, specifically in medical detection. AI has been widely used to gain more accurate detection results and decrease the burden on HC system. It can decrease the decision time associated with the detection process of traditional methods [40, 64]. Using MCDM can also assist researchers to leverage lessons learned through investigating past widespread disease events to predict who may be infected, where vaccination and treatment effort should be prioritized and how to limit the spread of infectious diseases in the future [67]. Meanwhile, awareness on COVID-19 and its early detection and proper treatment can help contain the disease.

## Recommendations

**Fig. 10:**
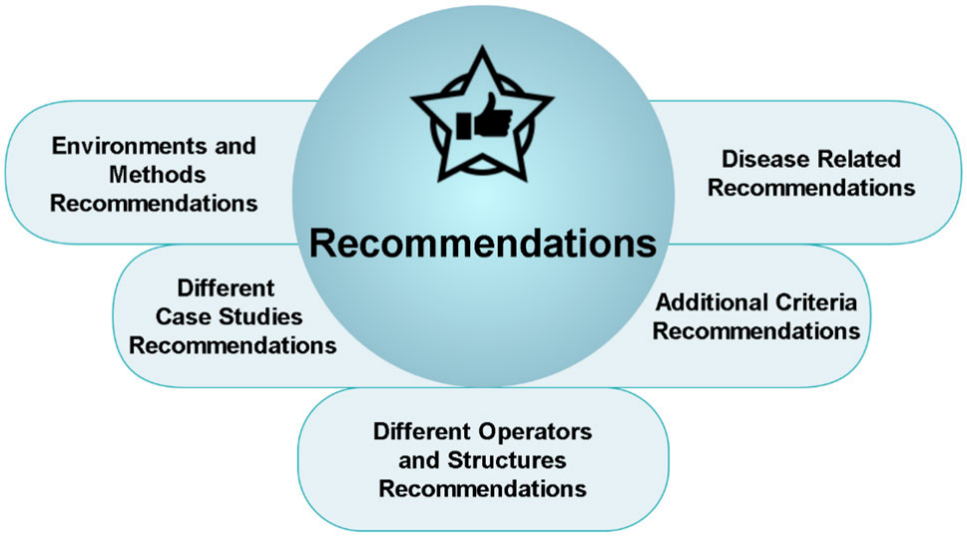
Main recommendations

Literature provides numerous recommendations, and components of these recommendations are technical related and the others are related to medical cases. This section presents the main recommendations in literature as seen in Fig. 10.

### Environments and methods recommendations

Various studies are recommended to be extended to other MCDM methods [53–55, 68, 71, 79, 80, 83, 84]. For example, a recommendation is to deal with the issue of uncertainty in HC setting prioritization using fuzzy, intuitionistic fuzzy, hesitant fuzzy, neutrosophic fuzzy, or probabilistic information [71]. Moreover, a study for evaluating strategies on enhancing HC resilience to combat COVID-19 recommended to be extended to other MCDM methods (e.g., AHP, TOPSIS and VIKOR) [39, 72]. The FDOSM and FWZIC approaches are recommended to be employed in the different COVID-19 cases owing to their promising results in terms of accuracy and consistency of their weighting and ranking [66]. Fuzzy MCDM techniques are recommended to be utilized to overcome the uncertainty problem for COVID-19 cast studies [41]. Different studies have recommended to be extended to other fuzzy environments [62, 73], such as interval-valued spherical fuzzy environments [37]. Others recommended hesitant fuzzy sets [37]. Additionally, a study on COVID-19 has recommended to be extended to probabilistic linguistic term, bipolar CF, q-rung orthopair fuzzy and neutrosophic sets [60]. Neutrosophic, intuitionistic and interval-valued and interval type-2 hesitant sets are recommended to overcome the uncertainty limitation [48]. T-spherical fuzzy, plithogenic hypersoft and complex neutrosophic hesitant fuzzy sets are recommended as well [44].

### Different case studies recommendations

Literature recommends the proposed methods to be applied in different fields [39, 53], such as medical diagnosis [37] and temporary hospital locations during the COVID-19 pandemic [52]. Furthermore, a method is proposed for selecting anti-virus mask to extend their proposed method into other HC problems, such as brain hemorrhage, disease recognition and medical diagnosis [43].

### Different operators and structure recommendations

Literature recommends the use of different DM techniques operators [39] and structures [39]. A study recommended different structures to be established under the Pythagorean fuzzy sets, such as ordered, algebraic and topological structures [42]. Moreover, the fuzzy symmetry concept is recommended to be utilized for antivirus mask selection to give more uncertain linguistic set applications in symmetry [44], Subset neighborhood rough sets [85], dense sets [86], and other rough sets environments [87]. Information-aggregation operators are also recommended to be utilized and investigated under spherical normal fuzzy environment, such as spherical normal fuzzy interaction operators and spherical normal fuzzy Hamy mean operators [43].

### Additional criteria recommendations

Literature recommends different parameters or criteria to be added for further analysis. For example, a recommendation is to add more symptoms and larger data sets to identify COVID-19 [36]. Another study recommends to add more preventive measures for COVID-19 spread assessment [68]. Additional criteria are likewise recommended to be added to the decision matrix of the study to support their evaluation and benchmarking in selecting the best methodology of diagnosing COVID-19 [40]. New criteria or sub-criteria are likewise recommended to be added to improve the COVID-19 regional safety assessment using the MCDM methods [41]. To improve the COVID-19 vulnerability geospatial modeling using fuzzy MCDM methods, age distribution, ethnic factors, and climatic factors are recommended to be added as parameters [83].

### Diseases-related recommendations

For COVID-19 vaccination accessibility analysis, a recommendation is to include the coordination and planning of procurement activities, storage, distribution, and monitoring of vaccine-dose availability [45]. Another study suggested to consider additional criteria and facts to improve the developed COVID-19 vulnerability map to accurately predict future COVID-19 outbreaks [83]. Another recommendation is to classify COVID-19 vaccine priority groups on the basis of availability, safety, delivery and cost [46]. Furthermore, it is recommended to analyze more COVID-19 vaccines and provide additional criteria to the MCDM approaches on the basis of individual priorities [77]. Ranking hospitals during the COVID-19 pandemic is recommended to identify more criteria, including the quality of equipment and hospital staff satisfaction, along with the quality of services and patients satisfaction [55]. To assure care for many people during COVID-19 pandemic, further analysis is suggested by including different variables, such as safe nursing homes, safe mask, safe homes, location for quarantine centers, and isolation planning, as well as an epidemic controlling model and ICU bed augmentation model [73]. Lastly, prioritizing COVID-19 vaccine dose recipients is recommended to present and process a large-scale data set of COVID-19 vaccine recipients by considering all probabilities frequently augmented for each alternative.

## Critical analysis

MCDM is one of the most intriguing and helpful tools that aided, and continues to aid, during the COVID-19 pandemic. Regardless of where it is applied, MCDM pioneered in many aspects of literature, including social, science and even medical research. Among the most important aspects is the medical application of MCDM-related research in COVID-19 settings. In this review, a comprehensive analysis is conducted from taxonomy analysis and discussion for main literature aspects. Some of these aspects are challenges, motivations, and recommendations related to COVID-19 directly (as a disease) or to the integration of MCDM with COVID-19 cases (application level). Notably, the authors report back on their previous peer’s issues, which hinder previous research effort. An important aspect is that the authors reflect their own point of view and implications based on the analysis conducted. For the current study, numerous attempts have been made to address many of the current challenges, such as those related to the MCDM techniques, COVID-19 and those that combine them. However, minimal focus has been directed to the security of medical data used in the process. Medical data are generally sensitive because they involve confidential information on patients’ treatment and should not be disclosed. These medical data are occasionally shared between hospitals and patients for medical decisions, such as the prioritization of patients or even assigning them for treatment.

Based on our taxonomy analysis, MCDM is used in the medical context for many medical-related cases (diagnosis models, safety aspects, hospital related aspects, and treatment). All aforementioned cases have their own medical data, whether patient-, treatment- or even hospital related. These medical data are sensitive, and sharing them between hospitals even for treatment purposes requires approval. During the COVID-19 pandemic, a difficult undertaking is to continue sharing medical data between hospitals while maintaining privacy and security. This endeavor poses a significant challenge because MCDM cannot work without actual data, and that data are transferred and prone to online misconduct, such as hacking or any malicious activities, resulting in the inseparable nature of hospitals in using online hospital networks [88]. For example, if hackers gain access to the hospital network and found patient data, they would have the ability to alter the course of treatment of patients and jeopardize the entire treatment process. Consequently, death may ensue in severe cases. Therefore, hospital networks should keep its performance in distributing data within more than one hospital to provide HC services and maintain data security and privacy, while important findings related to patient treatment are still generated.

Motivated by the preceding fact, the academic literature has presented a concept called “federated learning” (FL), in which a distributed set of machine processes are trained on huge medical data [89]. This concept focuses more on bringing the code to the data rather than having the data transferred into the code or processing, thereby reducing privacy and security issues. This approach has been recently introduced but is also already extensively utilized in ML research for various cases, the most important of which is medical-related. This situation is apparent because of the influx of patients and their medical data during the COVID-19 pandemic. FL is considered an exact-fit approach that only applies distributed learning to fit the privacy data challenge. FL has been applied in treatment-related studies for COVID-19, including [90], which presents FL to build prediction models of mortality for COVID-19 patients based on their e-health records (EHR). Moreover, Ref. [91] used FL for developing a diagnostic model for SARS-COV-2 to provide a robust model that can serve medical centers without sharing patient data. Evidently, FL is widely utilized in the context of ML, but patient medical data during crisis related times and for treatment related purposes such as the one with COVID-19 should also be studied. Given the advantages of FL, we propose to integrate it as a novel solution with decision science, particularly MCDM for different applications, such as treatment distribution. The following sections detail a proposed methodology on the utilization of FL as a novel application with MCDM for COVID-19 treatment distribution. All major steps to be considered will be detailed along with their rationale and how to apply it.

## Proposed methodology

**Fig. 11:**
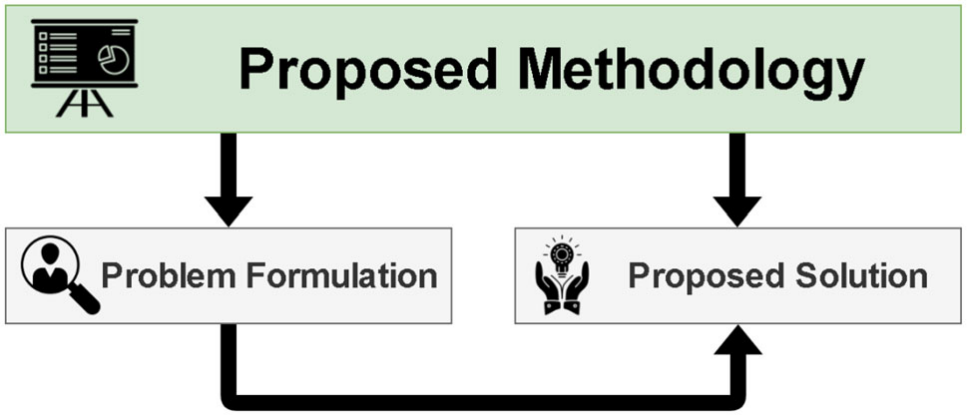
Proposed methodology

This section discusses the proposed methodology for integrating FL fundamentals in the context of MCDM for treatment purposes. Four subsections are likewise presented, in which the first discusses the formulation of the MCDM problem requiring FL (Fig. 11).

### Problem formulation

**Fig. 12:**
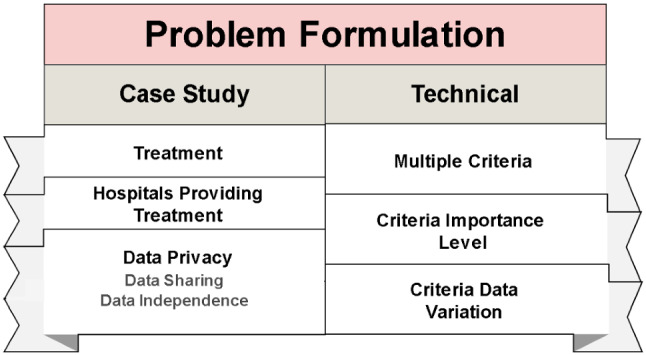
Problem formulation

We discuss the formulation of the problem, leading to the integration of federated MCDM in the proposed case study. Two main challenges are discussed, i.e., those related to the case study and those related to the technical aspect. Figure 11 illustrates the main sections of problem formulation (Fig. 12).

#### Case study (treatment)

No specific treatment is available at the start of the COVID-19 pandemic. Hence, numerous research endeavors have emerged to assist, including the vaccine, and others. In spite of the substantial benefits provided by previous treatment effort, the introduction of monoclonal antibodies (mAb’s) is presented as a treatment solution with more feasibility [92]. This type of treatment has long been recognized for giving immediate, passive immunity to individuals and help decrease disease symptoms and progression [93]. Typically, it is better if individuals are vaccinated prior to being infected, but the virus typically moves faster than the vaccination pace. In this regard, and given the impossibility of preventing infection, symptoms should be reduced to certain levels [94], and mAb’s is a pioneer in this aspect [93, 95]. Despite the suitability of mAb’s as treatment, if its distribution effort is not as quick as the virus spread, many people’s health would worsen and severely influence patients’ quality of life, even the economy [96]. This issue is worthy of consideration because it is a normal situation distribution problem and also because of the limited supply of this treatment, thereby raising a significant challenge regarding who should have it and the order of receiving it. Accordingly, a global interest is generated on the distribution of SARS-CoV-2 medicinal treatments [96]. The distribution significance is observant for treatment and is also reported for various medical and COVID-19-related cases, such as vaccine distribution**.** Therefore, this problem is worthy of investigation because there are factors that may play a role in the distribution process that differ from one country to another [97]. This type of treatment cannot be given or distributed without medical supervision. Furthermore, this supervision is constantly directed at medical centers or hospitals, making them an integral challenging part in the process.

#### Case study (hospitals providing treatment)

While addressing the problem of mAb treatment distribution, hospitals play a significant role in the process. Hospitals often administer their treatment distribution in such pandemic times. They rely on their network infrastructure to process the huge amount of patient data and to keep their channel of communication open with other hospitals, particularly if they are placed in different geographical locations [88]. These types of networking services are introduced to improve the efficiency of HC delivery and share specialized medical services and specialties [98]. Moreover, such a noble process does not come without risks, which are in the form of patient data privacy and security challenges [99]. They are important in usual circumstances and when they are used for SARS-CoV-2 treatment availability in the distribution hospitals. Therefore, this issue is a significant challenge to be considered. The following section discusses the data-privacy challenge for SARS-CoV-2 medical treatment and distribution.

#### Case study (data privacy)

Data sharing and privacy are among the most studied topics in many academic fields. They present a significant aspect for consideration, and they have a huge set of significantly different data cases, such as those for patient medical data. This issue is presented in this study’s context because medical data are often shared for treatment purposes between hospitals using network and telehealth services [100]. There are serious security breaches that may endanger patients’ health severely should hackers successfully hack medical data {MEDICA, 2020 #85}. These breaches may cause unprecedented alteration in patient treatment regimen, thereby potentially causing serious concerns. Therefore, the confidentiality of patient data must be safeguarded against unauthorized use and disclosure to third parties without patient consent. To safeguard patient data, two data privacy subchallenges are worthy of addressing: data sharing and independence. These aspects are reported in the following subsections.

*Data sharing* Data sharing among hospitals must be ensured as part of data-privacy protection. This process is mostly needed during difficult times, such as during the COVID-19 pandemic, when data are shared between medical institutions and researchers globally to develop good counter-strategies and treatment. This issue is worthy of consideration, particularly in a centralized system to establish a secure communication path between hospitals.

*Data independence* Another subchallenge for data privacy is data independence. This issue is part of data handling when used alongside database-management system (DBMS). DBMS enables users to alter data definitions and structure without influencing hardware and software. This practice enables various users to access and process data for different purposes. This issue is also apparent in a hospital setting, where DBMS are used to manage patient medical data, thereby assisting in making medical decisions. However, each hospital has its own DBMS format for storing and processing patient medical data. This situation presents data-privacy issues, in which no single representation of data distribution of patients exists [101]. This issue plays significant role in the privacy challenge for each hospital and is worthy of pursuing.

A summary of all previously discussed challenges and issues associated with the case study is presented as follows.

Statement 1: Providing mAb treatment to COVID-19 patients is challenging because of limited resources and different patient priority groups.

Statement 2: Using patient medical data for mAb treatment purposes among different hospital networks while maintaining privacy is a challenging task.

Statement 3: Maintaining patient medical data privacy for mAb treatment is challenging when data sharing is not secured and data used between hospitals are not unified.

The three statements present valid challenging problems that should be addressed in future research. Upcoming issues for future research in terms of technical aspects should be discussed to show how these problems are solved. Accordingly, the proposed problem definition subsection discusses the technical-problem definition.

#### Technical issues (prioritization)

For the technical challenges, the distribution of treatment should be prioritized. Hence, it is important to know which factors are influencing and to which extent this prioritization process. According to the National Institutes of Health, treatment guidelines suggest the use of Anti-SARS-CoV-2 for treating mild-to-moderate symptoms and SARS-CoV-2 infection PEP for those with severe symptoms [92]. For the ones from the last severe category, differences exist between patients who have been fully vaccinated because they are less likely to be infected again compared with patients who are partially vaccinated or even patients fully vaccinated but are not expected to generate a significant immunological response to the vaccine [102, 103]. This situation presents an issue of which patients may benefit the most from the treatment when treatment supply restrictions render impossible the treatment of all eligible patients. Therefore, triage becomes essential [104]. When this issue is presented, hospitals should utilize clinical discretion when prioritizing the use of anti-SARS-CoV-2 mAb’s for treatment or PEP in a particular context. This situation presents a multi-attribute decision challenge. Given the variety of treatment decision criteria, their importance level and data variation affect the prioritization main technical challenge. Details of the three subchallenges are presented in the following subsection.

*Multiple criteria* The issue of multiple evaluation criteria occurs when patient prioritization to receive mAb medical treatment for COVID-19 is subjected to different aspects, which influence the prioritization process. Examples of these aspects are (1) age, (2) hypertension, (3) cardiovascular disease, (4) heart diseases, (5) chronic respiratory disease, (6) obesity body mass index, (7) immunosuppressive disease, (8) pregnancy, and COVID-19 severity. The different aspects are referred to as criteria from literature and they are used to determine the urgency of a patient in the process of treatment distribution. Some of these criteria hold more importance than others, which are not particularly applicable in normal life situations. However, when they are presented to patients who are about to receive mAb medical treatment, some of these criteria are more important than others, which affect the patient prioritization. Details of the criterion importance are discussed in the next sub-problem.

*Criterion-importance level* Criterion importance in the context of MCDM refers to how significantly these criteria can affect the treatment-distribution process. For example, two patients require mAb treatment but the first one is 70 years old and the other is 35 years old. The first one suffers from diabetes and the second one suffers from kidney failure. According to the criterion importance, the second patient received treatment even though he is younger. The reason is that his other criterion of having kidney disease outweighs the diabetes criteria and the old age of the first patient. This example shows how different criteria with different importance significantly affect the prioritization process. The importance level for these criteria can either be conducted subjectively by decision makers (experts) [105, 106] or objectively via the fixed-weight method [107]. Regardless of which of them is the most qualified, they are both presented to address the main issue; In other words, which among the criteria is more important than the others. However, even if these criteria are given different importance levels by the previous means, l some medical data, particularly the ones in mAb’s, can be varied in their representation. For example, age is represented by the number of years, while having diabetes or kidney disease is a Yes/No question. This situation presents a data variation issue that also affects the prioritization process for mAb medical treatment. Details of the data variation issue are presented in the next section.

*Criterion data variation* The data-variation issue refers to the different representations of data criteria from literature. Variations listed in the academic literature have been generally concerned with the ranking process in eligible high-risk patients [108–111]. Moreover, data variations can be considered a special scenario to offer a clear discussion and analysis of this concern. For example, a scenario may occur as a goal with maximum representation, which is related to the distribution matrix for patients and their different criteria. In this context, the scenario can be observed when the maximization values of the criteria (↑) affect the data of the alternatives (i.e., high, higher and highest levels), in which the aim is to achieve a better selection process. Therefore, the prioritization process involves a simultaneous consideration of multicriterion matrices of the treatment-distribution process for eligible high-risk patients, in which different maximization goal scenarios are represented by the varying high, higher and highest levels that generate various data. A summary of all previously discussed technical challenges and issues is presented as follows.

**Statement 4:** *Distributing mAb treatment to COVID-19 patients is challenging because of the criterion tube considered in the decision process.*

**Statement 5:** *Distributing mAb treatment to COVID-19 patients is challenging because of the importance level for the prioritization decision criteria.*

**Statement 6:** *Distributing mAb treatment to COVID-19 patients is challenging because of the variety of the criterion tube considered in the decision process.*

### Proposed solution

**Fig. 13:**
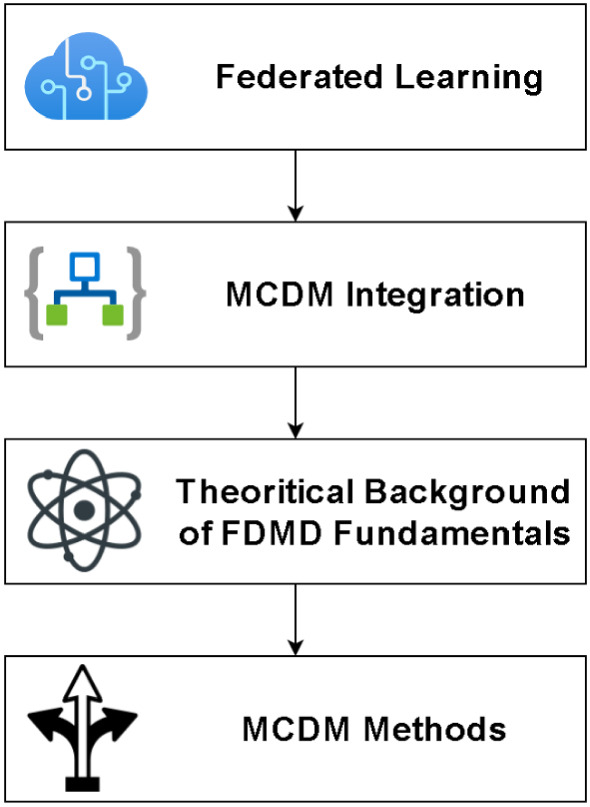
Proposed solution flow

This subsection discusses the federated MCDM methodology used as proposed solution. Figure 13 shows the flow of the method.

#### Federated learning

FL is a new application of AI, in which data training and learning occur on decentralized serve, and the findings are taken into the edge or on-device. FL is a popular trending topic, even though it is still in its early stages. FL works by distributing ML training models on data and training and takes learning to the edge or on-device large corpus of distributed data, according to the definition in Ref. [89]. It has been utilized in various medical applications, and its integration during the COVID-19 pandemic is apparent in different areas of research. Several studies have been conducted in this regard. Ref. [90] used FL to build prediction models of mortality in SARS-COV-2 patients based on their e-health records (EHR). Reference [91] adopted FL for developing a diagnostic model for SARS-COV-2 to provide a robust model that can serve as much as possible medical centers without sharing patient data. Reference [112] proposed an innovative collaborative model that allows numerous city Digital Twin (city DT) in the same region to immediately communicate the local plan and status for crisis management. Reference [113] that proposed a novel framework for early warning of SARS-COV-2 using crowd source and federated surveillance models that protect the privacy and allow social participants who do not have mutual trust to share the verified surveillance resources and blend their surveillance solutions. By considering how FL works, it enables local hospitals to learn collaboratively without the need for patient data sharing with a centralized or medical central server. In other words, patients’ data located and processed in the hospital. Nevertheless, FL has some medical applications, such as for COVID-19. However, most studies have utilized it in ML or deep-learning context. To the best of the author’s knowledge, no study has utilized it for mAb treatment distribution as prioritization challenge. Accordingly, addressing this challenge requires decision science. The next section describes MCDM and its potential as a proposed solution.

#### MCDM integration

MCDM is defined as a decision-theory extension covering any decision with multiple objectives. It is a branch of science long recognized for its capability in addressing prioritization issues when several conflicting criteria are to be considered [65]. It provides immense benefits not only in theoretical research but also in many applied research cases, such as COVID-19 mAb treatment prioritization challenges. MCDM in literature has been used in different cases to prioritize COVID-19 treatments. For example, Ref. [63] proposed an intelligent framework on the basis of the MCDM context’s success in handling the patients’ prioritization issue over distribution hospitals networking for the transfusion of efficient CP from donor to the most critical SARS-COV-2 patients. In spite of the aforementioned medical studies, MCDM has only handled prioritization issues in normal settings, where no data challenges in terms if privacy are considered. Therefore, it having MCDM integrated as a supporting tool to FL is warranted in mAb treatment distribution and prioritization challenges. To the best of our knowledge, it has not been applied previously in such a context, so it is a noteworthy research focus. Therefore, we call for the formulation of a new federated fundamental concept called federated DM distributor (FDMD) to overcome both challenges, thereby ensuring the privacy of SARS-COV-2 health data and prioritizing the anti-SARS-CoV-2 mAb recipients within distribution hospitals.

#### Theoretical background of the FDMD fundamentals

This solution enables hospitals to collaboratively learn to prioritize patients without data exchange/sharing with a centralized medical center server. Hence, FDMD should comprise two key sequence processes, namely, a patient-prioritization process that uses the hospital’s local decision matrix (a unique decision matrix for each hospital). Moreover, the global distribution transmission process ensures privacy protection and equitable distribution across all hospitals concurrently. The first process occurs using representative data (abstract data) obtained from local decision matrices. This step ensures patient data privacy, but the issue of patients’ prioritization process continues to be confronted with other challenging issues (e.g., multi criteria, data variation, criterion weighting). Accordingly, we need to formulate an MCDM methodology that can address the aforementioned issue. The following subsection presents a short review of the most used MCDM techniques with their pros and cons and best suited for the proposed FDMD.

#### MCDM methods

Several MCDM methods have been used in literature for variety of decision problem cases. MCDM methods have been categorized as either mathematical or human approaches. Among these methods is a simple additive weighting (SAW), which obtains the weighted sum of the performance ratings of each alternative overall attribute [114, 115]. It comprises two basic steps: the scale of the values of all attributes to make them comparable and the sum of the values of all attributes for each alternative [115]. Another method is hierarchical adaptive weighting (HAW), which is one of the earliest and possibly the simplest MCDM technique. WSM allows the comparison of the alternatives by assigning scores, and using these scores thereafter to generate standard values for the alternatives under consideration. Another method is weighted-product method, which presents numerous similarities with WSM but different in terms of the main mathematical operation. The last technique is TOPSIS, which is based on the concept that the ideal alternative has the best level for all attributes, but the negative ideal is the one with all of the worst attribute values [116, 117]. Evidently, MCDM and its techniques cover a wide area of research. While determining which of these techniques can be suitable for the FDMD concept, TOPSIS evidently has the best fit because of its nature with positive and negative ideals. This characteristic enables the creation of numerous matrices distributed between local federated hospital clients and central servers. However, the classical version of the TOPSIS method has some weaknesses: (1) insensitive to small values [118], (2) distorts the original information [119], (3) ranks reversal flaws [120], and (4) relatively small distance between the positive and negative ideal solutions [121–123]. Therefore, an improvement in TOPSIS is considerably warranted. The following subsection explains the improvement of TOPSIS and how it can be used as an FDMD concept.

**Step 1: TOPSIS as FDMD.**

Literature has indicated many improved versions of TOPSIS to overcome the classical TOPSIS flaws [119, 122–129]. For the same purpose, Ref. [130] developed classical TOPSIS in terms of improving positive and negative ideal solutions, as well as the closeness degree formula to avoid the possibility of rank reversal. FDMD is an iterative prioritization process among hospitals, including periodically admitting or discharge patients (i.e., increasing or decreasing in the alternatives) at each hospital for a given time, thereby causing a rank-reversal issue. Improved TOPSIS proposed by [130] entails the use of the efficacy coefficient method (ECM)) to avoid a potential rank reversal when absolute ideal solutions used is adopted in this study for prioritization. In return for protecting patients’ privacy during the prioritization process, the adopted TOPSIS [130] is utilized with the federated fundamental concept to introduce federated TOPSIS (F-TOPSIS). The main contribution of F-TOPSIS is the unification of the positive and negative ideal vectors that compute at the server side and provided to all local machines, with the preliminary settings. Meanwhile, each local machine uses it for the local prioritization process and sent back to the server for combining and computing the global prioritization ranks for each alternative (i.e., patients) over the LM networking. This sequence synchronized process of F-TOPSIS that implemented between CFS, and LM consist of the following eight steps.

**Step 1: Proposed the dynamic decision matrix (DDM) locally.**

For each local machine, DDM is constructed on the basis of the crossover of the mAb treatment distribution criteria and eligible patients, as shown in Table 1 using Eq. (1).

**Table 1:** DDM matrix

Alternatives	C1	C2	–	–	Cm
A1	C1/A1	C2/A1	–	–	Cm/A1
A2	C1/A2	C2/A2	–	–	Cm/A2
A3	C1/A3	C2/A3	–	–	Cm/A3
	–	–	–	–	–
	–	–	–	–	–
An	C1/An	C2/An	–	–	Cm/An

1 $${{{a}}^{{k}}}_{{ij}} \quad {\rm where } \, i = 1 \dots {n},{ j }= 1\dots {m \, {\rm and} \, k }= 1\dots {K}$$

${K}$: represent the number of the local machine.

${n}$: represent the number of alternatives.

${m}$: represent the number of criteria.

**Step 2: Allocation of DDM values locally**.

This step includes the allocation of each alternative related to each criterion using Eq. (2).

2 $${{a}}^{{k}} ={{{a}}^{{k}}}_{{ij}}, \quad {\rm where }\, i= 1 \dots {n},{ j }= 1\dots {m \, {\rm and} \, k }= 1\dots {K}$$

${K}$: represent the number of the local hospital.

${n}$: represent the number of patients.

${m}$: represent the number of criteria.

**Step 3: Unified local positive and negative vectors at CFS.**

In this step, positive and negative ideal vectors are determined for each registered LM and passed to the federated central server for unifying process of the positive and negative ideal values using Eqs. (3) and (4) [130]. The equation generalized for all ${{{a}}^{{k}}}_{{j}}^{+}$ and ${{{a}}^{{k}}}_{{j}}^{-}$ for all LM.

3 $$\left\{\begin{array}{c}{{{a}}^{{k}}}_{{j}}^{+}=\left\{\underset{1\le {i}\le {n}}{{\rm max}} \, {{{a}}^{{k}}}_{{ij}} \, \mid {j}\in {{j}}^{+},\underset{1\le {i}\le {n}}{{\rm min}} \, {{{a}}^{{k}}}_{{ij}} \, \mid {j}\in {{j}}^{-}\right\}\\ {{{a}}^{{k}}}_{{j}}^{-}=\left\{\underset{1\le {i}\le {n}}{{\rm min}} \, {{{a}}^{{k}}}_{{ij}}\mid {j}\in {{j}}^{+},\underset{1\le {i}\le {n}}{{\rm max}} \, {{{a}}^{{k}}}_{{ij}}\mid {j}\in {{j}}^{-}\right\}\end{array}\right.$$

4 $$\left\{\begin{array}{c}{{{a}}^{{g}}}_{{j}}^{+}=\left\{\underset{1\le {k}\le {K}}{{\rm max}} \, {{{a}}^{{k}}}_{{j}}^{+} \, \mid {j}\in {{j}}^{+},\underset{1\le {k}\le {K}}{{\rm min}} \, {{{a}}^{{k}}}_{{j}}^{+} \, \mid {j}\in {{j}}^{-}\right\}\\ {{{a}}^{{g}}}_{{j}}^{-}=\left\{\underset{1\le {k}\le {K}}{{\rm min}} \, {{{a}}^{{k}}}_{{j}}^{-}\mid {j}\in {{j}}^{+},\underset{1\le {k}\le {K}}{{\rm max}} \, {{{a}}^{{k}}}_{{j}}^{-} \mid {j}\in {{j}}^{-}\right\}\end{array}\right.$$

Equation (4) generalized the ${{{a}}^{{k}}}_{{j}}^{+}$ and ${{{a}}^{{k}}}_{{j}}^{-}$ for all LMs in the federate negative and positive ideal vectors. Specifically, ${{{a}}^{{g}}}_{{j}}^{+}$ is the maximum value over all local maximum values and ${{{a}}^{{g}}}_{{j}}^{-}$ is the minimum value over all local minimum values.

**Step 4: Normalization.** In this step, the decision matrix for each local machine is normalized using Eq. (5) [130] based on the federated positive and negative ideal values and $\upbeta$ interval value sent by CFS.

5 $$\begin{aligned} & {{{b}}^{{k}}}_{{ij}}=\frac{{{{a}}^{{k}}}_{{ij}}-{{{a}}^{{g}}}_{{j}}^{+}}{{{{a}}^{{g}}}_{{j}}^{+}-{{{a}}^{{g}}}_{{j}}^{-}}\times\upbeta +\left(1-\upbeta \right),\\ & \quad {\rm where } \, 0<\upbeta <1{\rm and} \, k=1,\dots ,{K} \end{aligned}$$

**Step 5: Application of external weight values.** In this step, weights of each used criterion will be applied to the normalized decision matrix at each LM, using Eq. (6) [130].

6 $${{{y}}^{{k}}}_{{ij}}={{{b}}^{{k}}}_{{ij}}\cdot {\upomega }_{{j}}, \quad {\rm where }\, i=1,\dots ,{m};{j}=1,\dots ,{n}$$

**Step 6: Closeness determination.** This step includes defining the negative and positive using Eq. (7) [130] at LM.

7 $${{y}}^{*}=[{1,1},\dots ,1{]}_{{n}}^{{\rm T}}, {{y}}_{*}=({0,0},\dots ,0{)}_{{n}}^{{\rm T}}$$

**Step 7: Ranking the alternatives.** This step is the last one, in which alternative ranking is computed locally using LM and combine and sort globally at server side. At LM, the local score at each alternative compute using the project from each alternative ${{y}}_{{i}}$ to positive ideal solution y* using Eq. (8) [130].

8 $${{Pr}}^{{k}}\left({{{y}}^{{k}}}_{{ij}}\right) =\frac{\sum_{{j}=1}^{{n}} {{y}}_{{j}}^{*}{{{y}}^{{k}}}_{{ij}}}{\sqrt{\sum_{{j}=1}^{{n}} {\left({{y}}_{{j}}^{*}\right)}^{2}}}$$

Lastly, the patient rank will set ${X }= \{{x}1,{ x}2, \dots ,{ xm}\}$ according to the value of ${{Pr}}^{{k}}\left({{{y}}^{{k}}}_{{ij}}\right)$ (${i }\in {M}$).

9 $${Pr }= {\bigcup_{{k }= 1...{K}} {Pr}}^{{k}}$$

Thereafter, global rank orders the alternative ${X }= \{{{x}}^{1},{{x}}^{2}, \dots , {{x}}^{{k}}\}$ according to the value of ${Pr}$.

In the FDMD sequence process, the criterion importance has a vital role in the final results. However, one of the main limitations of federated-TOPSIS is the criteria weight are required an external method for weighting the criteria. In the MCDM context, there are two ways to assign weights to the criteria: objectively and subjectively [131]. In the objective weighting methods, the importance of criteria is computed based on raw data. In such a situation, raw data changes affect the accuracy of the weight value and are required to share the private data of patients. By contrast, the subjective weighting methods represent the experts’ cumulative knowledge and their subjective opinions [132]. Many subjective weighting methods have been introduced in literature. The following subsection discusses the main ones and which is best suited for the case study of this research.

#### Weighting method

For the future treatment case studies, and according to the MCDM approach, different criteria should be presented from literature on medical guidelines. They are used in the prioritization/ranking process. However, each of these criteria differs in significance and impact on the overall treatment decision. Notably, many approaches are presented, such as AHP [63] and BWM [133], which presents a high rate of success in weighting criteria. However, the inconsistency in their weighing methods continues to be an unsolved issue. Addressing these issues is resolved by the FWZIC [134] method achieved zero inconsistency by precisely determining the weight values of each criterion. Moreover, it can determine the criterion importance in the DM process with the assistance of experts [135]. The following section explains the FWZIC methodology.

##### *Criterion weighting technique*

Based on literature, a net method called novel fuzzy-weighted zero-inconsistency (FWZIC) [136] is proposed and proven more robust in mitigating the short comings of the previous weighting techniques. FWZIC has been proven to provide weights for criteria with zero inconstancy. Thus, this method is more promising in weighting criteria for the vast cases of MCDM integration with COVID-19 cases. This method also comes in different versions with different fuzzy types. Here, we describe its main steps.

**Phase 1:** Criterion definition. In this phase, all the ready-to-be-utilized criteria are introduced to FWZIC after being evaluated and defined.

**Phase 2:** Structured expert judgment. In this phase, the criterion defined from previous step is evaluated for their importance level by panel of expert. These experts should be specialists with sufficient academic and research experience. Afterwards, a nomination process is established through the following steps.

Step 1 Expert identification. Experts are identified as those who have been or still involved in the subjects of the case study and regarded as knowledgeable by others.
Step 2 Expert selection. After completing expert identification, some of them are deemed most suitable for the case study. A minimum of four experts are needed in this stage. For the expert’s panel, they should be between 10 and 16. All experts from the previous stage are reached out via email to determine their availability and willingness to be considered potential experts for the panel.
Step 3 Developing evaluation form. The evaluation form used in the case study is developed in this stage for collecting experts’ consensus, and it undergoes reliability and validity check.
Step 4 Defining the importance level scale. All selected experts from previous step define the importance level for each criterion with the use of a five-point Likert scale.
Step 5 Converting linguistic scale to numerical scale. This step includes transforming all preference values are from subjective form into numerical form so they can be utilized in the analysis. Thus, the importance level for every criterion from each expert in the used Likert scale is converted into numerical scale, as shown in Table 2.

**Table 2:** Five-point Likert scale and equivalent numerical scale

Numerical scoring scale	Linguistic scoring scale
1 2 3 4 5	Not important Slight important Moderately important Important Very important

**Phase 3:** Building EDM. This step includes creating EDM with the main parts, which include the criteria and alternatives, as shown in Table 3.

**Table 3:** EDM

Experts	C1	C2	…	Cn
E1	Im (E1/C1)	Im (E1/C2)	…	Im (E1/Cn)
E2	Im (E2/C1)	Im (E2/C2)	…	Im (E2/Cn)
E3	Im (E3/C1)	Im (E3/C2)	…	Im (E3/Cn)
…	…	…	…	…
Em	Im (En/C1)	Im (En/C2)	…	Im (Em/Cn)

Im refer to the importance degree

The previous phase provides a definition for the list of selected experts and the preference of each expert within a single criterion. EDM is constructed in this step. The main parts of EDM are the decision criteria and alternatives.

**Phase 4:** Application of Fuzzy memberships.

FWZIC is a technique of MCDM used to measure the criteria weight based on human subjective assessment. The earliest version of this method developed with fuzzy to help reducing the uncertainty associated with the subjectivity of human decision makers. In this step, the main formulae to be used in FWZIC [137] are presented based on the triangular fuzzy numbers set. Below are the formulas of the selected fuzzy set:

- Fuzzy membership
  10 $${\mu A}\left({x}\right)= \left\{\begin{array}{ll}0 & {\rm if} \, x<a\\ \frac{{x}-{a}}{{b}-{a}}& {\rm if} \, a\le x\le b\\ \frac{{c}-{x}}{{c}-{b}} {\rm if} \, b\le x\le x\\ 0& {\rm if} \, x>c \end{array}\right.,{\rm where} \, a\le {b}\le {c}.$$
- Arithmetic operations
- Addition
  11 $$\widetilde{{x}}+\widetilde{{y}}=\hspace{0.17em}({{a}}_{1}\hspace{0.17em}+\hspace{0.17em}{{a}}_{2}, {{b}}_{1}\hspace{0.17em}+\hspace{0.17em}{{b}}_{2}, {{c}}_{1}\hspace{0.17em}+\hspace{0.17em}{{c}}_{2})$$
- Subtraction
  12 $$\widetilde{{x}}-\widetilde{{y}}\hspace{0.17em}=\hspace{0.17em}({{a}}_{1}\hspace{0.17em}-\hspace{0.17em}{{c}}_{2}, {{b}}_{1}\hspace{0.17em}-\hspace{0.17em}{{b}}_{2}, {{c}}_{1}\hspace{0.17em}-\hspace{0.17em}{{a}}_{2})$$
- Multiplication
  13 $$\widetilde{{x}}\times \widetilde{{y}}\cong ({{a}}_{1}{{a}}_{2},{{b}}_{1}{{b}}_{2},{{c}}_{1}{{c}}_{2})$$
- Division
  14 $$\widetilde{{x}}/\widetilde{{y}}\cong ({{a}}_{1}/{{c}}_{2}, {{b}}_{1}/{{b}}_{2}, {{c}}_{1}/{{a}}_{2})$$
- Division on crisp value
  15 $$\widetilde{{x}}/{\alpha }=({{a}}_{1}/{\alpha }, {{b}}_{1}/{\alpha },{{c}}_{1}/{\alpha })$$
- Defuzzification
  16 $$\frac{({a}+{b}+{c})}{3}$$

**Phase 5:** Final weight computing.

In this step, FWZIC technique applied with the identified fuzzy set-in Formulas 9 to 15 are used to measure the final weight. These steps can be compromised into three substeps.

i. The ratio of fuzzification data is computed using Eqs. (11) and (14).
ii. Average values are computed to find the final fuzzy values of the weight coefficients of the evaluation criteria${(\widetilde{{w}1},\widetilde{{w}2}, ...,\widetilde{{wn}})}^{{\rm T}}$, using Eq. (15).
iii. Defuzzification is conducted to determine the final weight. In particular, defuzzification methods are used to find the crisp weight value using Eq. (16) Prior to calculating the final values of the weight coefficients, the weight of importance of each criterion is assigned based on the sum of the weights of all criteria for the rescaling purpose applied in this stage.

## Conclusion

We conduct an SLR of several COVID-19 cases studies using the MCDM approach and obtain a final set of 32 selected studies (*n* = 32). These studies are taxonomized and categorized into different classes, namely, diagnosis (*n* = 6), safety (*n* = 11), hospital (*n* = 8), treatment (*n* = 4), and review (*n* = 3). Two aspects are identified in diagnosis class: (1) computerized and hospital-based tests and (2) regional assessment, masks and sanitizer toward the safety measures. For safety class, four topics are identified considering the linkage of MCDM with medical settings, which are regional assessment, sanitizers, mask selection and vaccine. For the hospital class, the discussion is made on the basis of the use of MCDM in medical settings for COVID-19 situation in term of hospital selection and admission, hospital services, and patient prioritization. Another two topics are discussed with the treatment class: medicine and plasma. Finally, the review articles that discuss MCDM studies in relation to COVID-19 applications. A state-of-the-art bibliographic analysis is presented based on R-tool and VOSviewer to explore annual scientific production, country scientific production, co-occurrence, and co-authorship. Moreover, different highlights including challenges (diseases and techniques), motivations (diagnosis, DM, HC resources, vaccines/treatment, and detection), and recommendations (other environments and methods, different case studies, different operators and structure, additional criteria, diseases) are analyzed. However, this study identifies research gaps for distributing mAb vaccine doses among different hospitals given the data-related issues, such as privacy, data type and being an MCDM issue given the difference priority groups eligible for the vaccine given the lack of resource. Lastly, a desirable solution comprising two main aspects is proposed. The first is involves integrating MCDM to address the distribution issue given the different priority groups and lack of vaccine resources. The second involves modifying the typical MCDM to make it comparable with the FL concept and address the issues associated with data sharing and privacy expected to occur during patient-information exchange among different hospitals for treatment. The proposed method in this research is based on TOPSIS. However, in the future, other MCDM techniques with normalization (i.e., VIKOR, MULTIMOORA, and MARCOS) and others can be integrated into the federated concept. Lastly, the security aspect can also be considered by integrating the future federated concept with more robust technologies such as blockchain between client and server to ensure multilevel privacy.

## Data Availability

Availability of data and material not applicable.
